# Engaging tuberculosis-affected people in investment decisions: applying a people-centered design approach in Viet Nam

**DOI:** 10.1186/s12889-026-29213-z

**Published:** 2026-08-27

**Authors:** Rachel Forse, Phuong-Tra Vu, Trang Nghiem, Luan Nguyen Quang Vo, Andrew James Codlin, Ngoc B. Pham, Ngan Huynh, Thanh Thi Nguyen, Luu Quang Vinh, Nguyen Thi Kim Ngan, Nguyen Thi Thuy, Pham Viet Phuong Khanh, Nguyen Duc Bang, Andrew Cross, Michael Wilson, Karolin Lüneburg, Montana Cherney, Tobias Silberzahn, Luong Van Dinh, Hoa Binh Nguyen, Lan Huu Nguyen, Ha Dang Thi Minh, Khai T. M. Nguyen, Tiep T. Hua, Jacqueline Huh, Kristi Sidney Annerstedt

**Affiliations:** 1 Friends for International TB Relief, Hanoi, Viet Nam; 2 Department of Global Public Health, Karolinska Institutet, Stockholm, Sweden; 3 Tuberculosis-affected Person, Hanoi, Viet Nam; 4 Stop TB Partnership, External Affairs and Strategic Initiatives, Geneva, Switzerland; 5 Centre for the AIDS Programme of Research in South Africa, University of KwaZulu-Natal, Durban, South Africa; 6 Independent Consultant, Munich, Germany; 7 A Healthier Future GmbH, Berlin, Germany; 8 National TB Programme, National Lung Hospital, Hanoi, Viet Nam; 9 Pham Ngoc Thach Hospital, Ho Chi Minh City, Viet Nam; 10 Can Tho Tuberculosis and Lung Hospital, Can Tho City, Viet Nam

**Keywords:** Community engagement, People-centered design, Re-imagining TB Care, Participatory methods, Resource allocation, Tuberculosis, Viet Nam, Social protection

## Abstract

**Background:**

Tuberculosis (TB) remains the leading cause of death from an infectious disease globally, yet financing for the TB response continues to fall far short of identified needs. Decisions about how resources are allocated are often made with limited engagement of people directly affected by TB, potentially overlooking lived experiences and community-defined priorities. This study was implemented in Viet Nam to align future TB investments with community-defined priorities and programmatic feasibility. This paper describes the process and its application to priority setting and intervention selection alongside national planning processes.

**Methods:**

This was a participatory co-design study guided by a people-centered design framework. The process integrated qualitative data collection and deliberative stakeholder engagement across three phases: Discover (qualitative interviews and focus groups to identify challenges), Define (stakeholder workshops to prioritize challenges and co-develop an evaluation framework), and Develop (structured prioritization assessment and selection of an intervention). In the Discover phase, 88 participants identified challenges across the TB care cascade. In the Define and Develop phases, decision-making was overseen by a diverse 21-member Leadership Group. Participants co-developed an evaluation framework across three domains (fit and desirability, potential impact, and feasibility), which was used to assess and rank potential investment opportunities. Facilitation strategies, including equal voting rights and anonymous ranking, were applied to promote equitable participation.

**Results:**

In the Discover phase, 15 key challenges across the TB care cascade were identified and reframed as opportunities for improvement. In the Define phase, stakeholders applied the evaluation framework to assess 19 innovation categories. Three intervention areas were shortlisted for detailed consideration: computer-aided detection software for chest X-ray interpretation, paper-free data systems, and social and financial protection. In the Develop phase, structured scoring and deliberation, including consideration of feasibility and the existing funding landscape, led to the selection of social and financial protection as the intervention for near-term investment and implementation.

**Conclusions:**

Participatory co-design enabled TB-affected people and health system stakeholders to directly contribute to early-stage investment prioritization within Viet Nam’s TB response. The selection of social and financial protection reflects both lived economic challenges and system-level considerations, highlighting the value of participatory approaches in identifying locally relevant priorities that may be underemphasized in conventional funding processes.

**Supplementary Information:**

The online version contains supplementary material available at https://doi.org/10.1186/s12889-026-29213-z.

## Background

Tuberculosis (TB) is the infectious disease that kills more people than any other, yet the global TB response historically has been underfunded, particularly in low- and middle-income countries (LMICs). In 2024, global financing for TB in LMICs amounted to USD 5.9 billion, which was approximately 27% of the USD 22 billion target set by Member States in the political declaration of the 2023 United Nations High-Level Meeting on TB [1, 2]. That year, TB funding was around one-third of that allocated for malaria and just 12% of the global funding provided for HIV [1]. These shortfalls were further exacerbated by unprecedented funding cuts in 2025, during which more than half of all external financing for the TB response was withdrawn [3, 4]. The persistence of this funding gap underscores the urgent need not only for new investments, but also for more transparent and inclusive processes to guide how increasingly scarce resources are allocated [5].

In the current landscape of constricted global health financing, questions about *who* participates in funding decisions and *how* priorities are set become increasingly important. Beyond identifying interventions that maximize public health impact and cost-efficiency, decision-making processes increasingly require scrutiny of their governance, inclusiveness, and accountability. Traditionally, public health investment decisions have involved a limited number of stakeholders, drawing on criteria such as disease burden, cost, and anticipated population-level benefits [6]. However, there is no global standard for establishing research or investment priorities, and the criteria applied are often subjective and weakly transparent [6, 7]. Although calls for greater inclusiveness through co-creation and co-design are increasing, these approaches rarely extend to the earliest stages of problem identification and prioritization, where public input could meaningfully shape research and implementation agendas [8, 9]. As a result, global and national decision-making often diverges from the lived experiences and priorities of affected populations, frontline healthcare workers, and community-based organizations [6, 10].

This disconnect is evident in TB programs, where planning and investment have historically been determined by politicians, administrators, policymakers, and technical experts, with limited opportunities for TB-affected people to influence which problems are prioritized or which solutions are pursued [11, 12]. To address this gap, initiatives such as the World Health Organization (WHO) Civil Society Task Force on TB and the Global Fund’s Country Coordinating Mechanisms have sought to expand community participation [13, 14]. While these mechanisms represent important progress, they often remain consultative in nature and have limited influence over early-stage priority setting and decisions about resource allocation. The lack of meaningful engagement with TB-affected people has been linked to weak advocacy and inadequate global support for TB [10, 15].

A variety of structured approaches have been developed to support priority setting in health systems and health research, including consensus-building methods, multi-criteria decision analysis, and preference elicitation techniques [16–20]. While these approaches provide systematic ways to evaluate options and weigh trade-offs, they are often expert-driven and involve limited engagement with affected communities. WHO conceptualizes community engagement along a continuum of five levels (inform, consult, involve, collaborate, and empower), illustrating how participation can vary in depth and influence over decision-making [21]. As a result, many approaches may insufficiently capture the lived experiences and priorities of people directly affected by health conditions such as TB.

One participatory approach that seeks to strengthen community engagement in priority setting and resource allocation is People-Centered Design (PCD). Drawing on principles from Design Thinking and Human-Centered Design, PCD emphasizes empathy, iterative problem solving, and attention to people’s lived experiences [22, 23]. In public health and social innovation contexts, PCD positions healthcare users, frontline providers, and community-based organizations as active contributors to problem definition, decision-making, and intervention development, rather than as passive recipients of services [24–27]. By combining deliberative methods with qualitative inquiry, PCD applies iterative, social science-informed approaches to support shared decision-making and the co-design of solutions within real-world systems, making it well-suited to complex health interventions and policy-relevant prioritization processes, and increasingly aligned with implementation science [28, 29]. Despite growing use across several public health domains, including HIV, applications of PCD in TB care remain limited [24, 30].

Although WHO-endorsed strategic planning approaches for TB programs are intended to engage a range of stakeholders, implementation varies across settings and tends to be consultative in nature, creating opportunities to strengthen meaningful participation throughout the planning process [31]. This gap is particularly pronounced in Viet Nam, where civil society engagement in health policy operates within state-managed structures with limited independent advocacy capacity, and domestic TB financing remains heavily constrained despite recent reforms in social health insurance [32–36].

This study was implemented alongside the development of the TB National Strategic Plan (NSP) 2026–2030, in collaboration with the National TB Programme (NTP). It represents the first systematic application of PCD within Viet Nam’s TB response. The objectives were twofold: [1] to apply a structured participatory co-design process, guided by a PCD framework, to inform priority setting alongside NSP development; and [2] to enable TB-affected people and health system stakeholders to directly select an intervention for near-term investment and implementation.

## Methods

### Context

Viet Nam is a Southeast Asian country that was reclassified from lower-middle- to upper-middle-income status in July 2026 [37]. With a population of approximately 99.5 million, it is characterized by considerable geographic and socioeconomic variation in health system capacity and access to services across provinces, municipalities, districts, and communes [38, 39]. The country ranks 12th among the 30 countries with the highest TB burden globally, yet TB detection remains low, with approximately 58% of estimated incident TB being notified and treated [40]. TB diagnosis and treatment are primarily initiated at the district level, with treatment follow-up managed at commune health stations within the four-tier public health system [41]. TB services are centrally coordinated by the NTP, which is housed within the National Lung Hospital [42]. The National Lung Hospital also serves as the Principal Recipient for the country’s TB grant from the Global Fund, with responsibility for financial management, procurement of TB commodities, programmatic reporting, and supervision of grant implementation through sub-recipients across the country [43]. However, under Viet Nam’s current decentralized health financing structure, provincial authorities allocate most domestic TB resources, and the NTP does not directly control the provincial TB budget [44].

Civil society engagement in health policy in Viet Nam remains limited and typically operates within state-managed structures with limited independent advocacy capacity [36]. Existing community-based organizations are primarily engaged in service delivery activities such as active case finding and treatment support; organized TB survivor networks remain limited [45].

Against this backdrop and to complement ongoing national planning processes and strengthen community engagement, the present study was conducted as part of the Re-imagining TB Care initiative, funded by Stop TB Partnership and the Korea International Cooperation Agency, to apply PCD methods to support participatory problem identification and intervention development within Viet Nam’s TB response [28]. The study was implemented by a core study team comprising staff from Friends for International TB Relief (FIT) and the Stop TB Partnership, with technical support from independent consultants.

### Study design

**Fig. 1:**
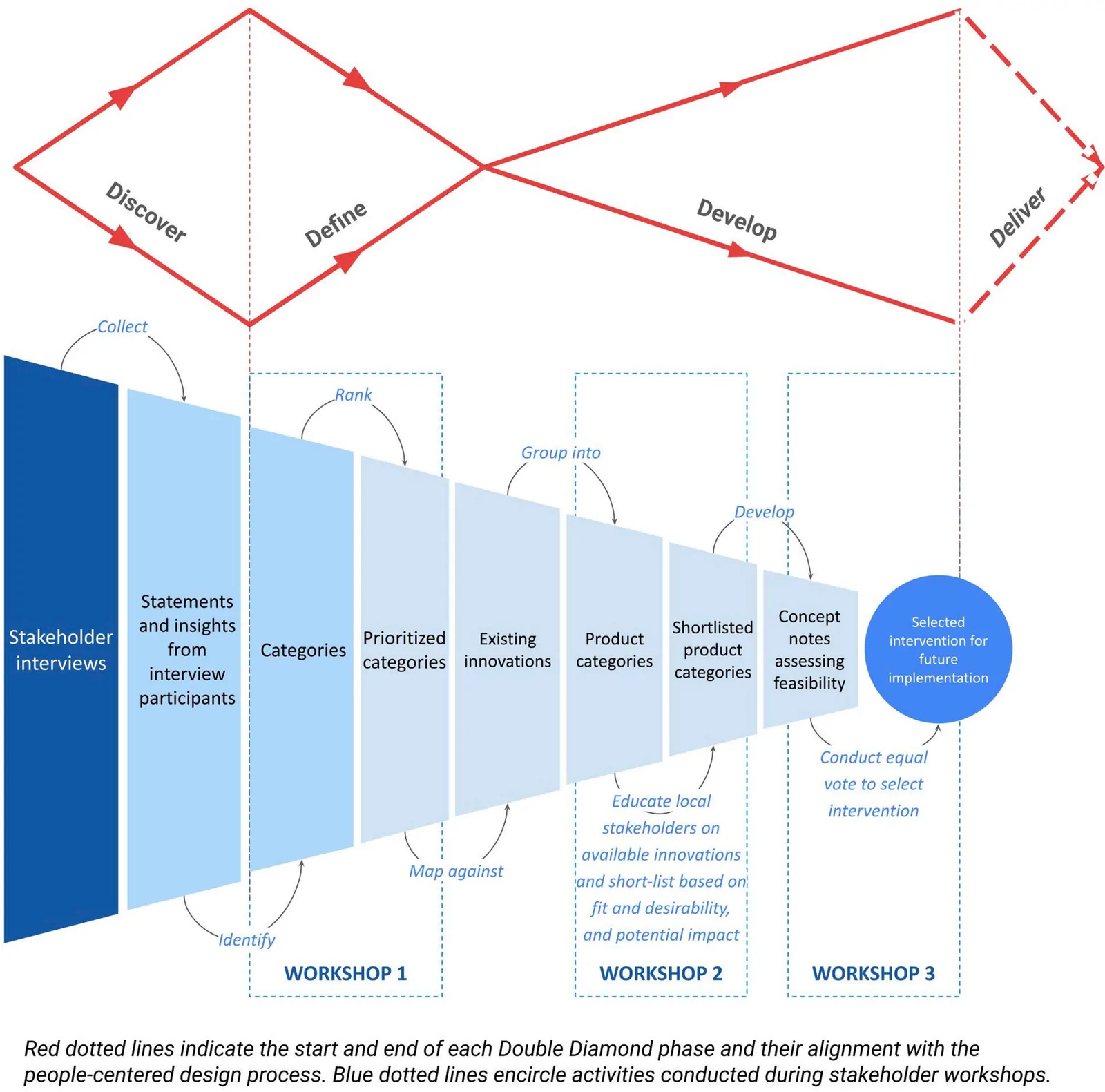
Integration of the Double Diamond framework with the people-centered design process in Viet Nam. Legend: Red dotted lines indicate the start and end of each Double Diamond phase and their alignment with the people-centered design process. Blue dotted lines encircle activities conducted during stakeholder workshops. Adapted from *The Double Diamond - Design Council* [46]

This was a participatory co-design study guided by a PCD framework. The study combined qualitative data collection with deliberative stakeholder engagement to move from consultation toward shared decision-making regarding future investment priorities. It was structured using the Double Diamond framework, which organizes the process into four stages: Discover, Define, Develop, and Deliver [26, 46]. The Discover phase involved qualitative interviews and focus group discussions to identify challenges across the TB care cascade. Findings were synthesized and opportunity areas were prioritized in the Define phase, and stakeholders participated in assessing and refining potential interventions in the Develop phase (Fig. 1). The Deliver phase (implementation) was outside the study scope. To support transparent reporting of stakeholder engagement, the Guidance for Reporting Involvement of Patients and the Public, Version 2 Short Form (GRIPP2-SF) checklist was used [47] (See Additional File 1).

### Discover

The PCD process began with stakeholder mapping and qualitative data collection to identify challenges across Viet Nam’s TB care cascade. Given the broad exploratory objective and need to capture diversity across stakeholder groups, geographic regions, and health system levels, a large sample of 80–100 participants was targeted [48]. Participants were recruited from five provinces representing the northern (Ha Noi, Hai Phong), central (Quang Nam), and southern (Ho Chi Minh City, Can Tho) regions of Viet Nam, encompassing a mix of urban and peri-urban settings with varying TB burden and service delivery contexts. Stakeholders were purposively sampled using maximum variation to include policymakers, TB program staff, healthcare providers (public, private, and community), NGOs/CSOs, and TB-affected individuals and communities [49, 50]. TB-affected people were defined as individuals with TB disease or TB infection (current or previous), their household members or caregivers. Potential participants were identified through the NTP, provincial TB programs, participating health facilities, and CSOs, with additional participants identified from stakeholder recommendations to ensure representation across groups and settings.

Eighty-nine identified stakeholders were approached, and 88 participated in 69 in-depth interviews and three focus group discussions. Individual interviews were conducted across all stakeholder groups, whereas the focus groups included TB-affected people and staff from two provincial lung hospitals. The final sample comprised 40 public-sector providers, five policymakers, four NGO/CSO representatives, six private-sector providers, and 33 TB-affected individuals from five provinces of Viet Nam, including Ha Noi (*n* = 34), Hai Phong (*n* = 11), Quang Nam (*n* = 18), Ho Chi Minh City (*n* = 12), and Can Tho (*n* = 13). Women represented 43.2% of participants, and 21.6% were aged 18–30 years. A summary of the qualitative sample is presented in Table 1.

**Table 1:** Demographics of qualitative interview participants

Characteristics	Healthcare providers	NGOs/CSOs	Policymakers	Private sector providers	TB-affected people	TOTAL
North	19	1	2	4	19	**45**
Center	11	1	2	0	4	**18**
South	10	2	1	2	10	**25**
**Total**	**40**	**4**	**5**	**6**	**33**	**88**
Female	21	1	2	3	11	**38**
Age 18–30	5	1	1	3	9	**19**
Age 31–45	18	1	1	2	13	**35**
Age 46–70	17	2	3	1	11	**34**

*Abbreviations*: *CSO* Civil society organization, *NGO* Non-governmental organization, *TB* Tuberculosis

Interview and topic guides were developed for each stakeholder group by the study team (Additional File 2), piloted, and revised prior to data collection. All participants were adults (aged ≥ 18 years), and written informed consent was obtained before participation. Participants received VND 500,000 (approximately USD 20.50) to compensate them for their time and travel.

Interviews and focus group discussions were conducted in Vietnamese by an experienced qualitative interviewer accompanied by a note taker. Data collection took place in a private room, typically within a healthcare facility. Sessions were audio-recorded and detailed field notes were taken and expanded immediately afterward. Audio recordings were reviewed to identify and transcribe illustrative excerpts relevant to the study objectives, which were translated into English as needed [51].

A modified six-step interview data management process, adapted from Halcomb and Davidson, was used to support rapid qualitative synthesis [52]. Rather than producing full verbatim transcripts, audio recordings and concurrent field notes were taken to ensure that both participants’ responses and contextual observations were captured. Immediately after each interview, the interviewer completed reflective journaling to document additional context and impressions. The audio and all documentation were reviewed to identify and extract data relevant to the study objectives. Extracted excerpts were entered into the Miro platform (*Miro visual workspace*, 2023), systematically reviewed, compared, and grouped into categories of challenges within the TB program through an iterative team-based process. Multiple members of the study team participated in categorization to ensure consistency and ground the data. A summary was developed, and categories were then organized across four predefined components of the TB care cascade: Screen, Diagnose, Treat, and Prevent. To support the transition from problem identification to solution generation within the participatory design process, each category was rephrased as a forward-looking design prompt framed as a “How might we…?” question. These prompts were developed to reflect the underlying challenge while enabling structured and solution-oriented discussion during subsequent stakeholder workshops. Table 2 provides an illustrative example of how data excerpts were categorized and translated into design prompts.

**Table 2:** Illustration of qualitative data categorization and translation into a design prompt

Example quotes	Category	Summary of category	TB care cascade step	Design prompt
*“Taking TB medication is just like taking medicine for a cold. The only problem is that there are too many drugs to take*,* each time 3 white and 3 pink pills*,* a whole handful of drugs.”* *—*TB survivor *“The treatment duration should be shortened. I hope that my patients will only have to have treatment for 1 month.”* *—* Healthcare provider *“Previously*,* my patients were accustomed to receiving their medication from the district TB unit*,* with visits required only once every 14 days or once a month. After services were shifted to ward-level units*,* they were required to visit every 7–10 days*,* which led to many complaints.”* *—* Healthcare provider	Improve TB treatment	There is a strong interest in better access to more tolerable, shorter drug regimens, fixed-dose combination medications and multi-month dosing.	Treat	*How might we increase access to more desirable and acceptable TB treatment regimens?*

*Abbreviations*: *TB* Tuberculosis

### Define

A decision-making working group, referred to as the Leadership Group, was established to guide the PCD process in the Define and Develop phases. Members were purposively selected to ensure representation across geographic regions, stakeholder groups, and levels of decision-making authority, to complement the NSP development process by integrating perspectives from both policy and practice. Selection criteria included: (1) representation from the northern, central, and southern regions of Viet Nam; (2) inclusion of people with lived experience of TB; and (3) involvement of stakeholders with direct roles in TB policy, planning, and service delivery. The group comprised 21 stakeholders from Viet Nam who had not participated in the earlier interviews, including people with lived experience of TB (*n* = 5), public and private healthcare providers (*n* = 4), policymakers (*n* = 10), and NGO/CSO representatives (*n* = 2). Members were selected from across Viet Nam, with participants from the northern (*n* = 9), central (*n* = 3), and southern (*n* = 9) regions. Women represented 42.9% of the group, and participants aged 18–30 years accounted for 14.3%. While individuals were sampled for specific experiences, the categories were not mutually exclusive; for example, one of the four healthcare providers also identified as a TB survivor. Table 3 presents a demographic breakdown of the Leadership Group.

**Table 3:** Demographics of Viet Nam’s leadership group

Characteristics	Healthcare providers	NGOs/CSOs	Policymakers	TB-affected people	Total
North	1	1	5	2	**9**
Center	1	0	1	1	**3**
South	2	1	4	2	**9**
**Total**	**4**	**2**	**10**	**5**	**21**
Female	1	2	2	4	**9**
Age 18–30	0	0	0	3	**3**
Age 31–45	2	2	2	1	**7**
Age 46–70	2	0	8	1	**11**

*Abbreviations*: *CSO* Civil society organization, *NGO* Non-governmental organization, *TB* Tuberculosis

Although policymakers represented the largest subgroup of the Leadership Group, facilitation strategies were intentionally designed to promote equitable participation and mitigate power imbalances. These included rotating facilitation roles, encouraging initial input from TB-affected participants, and using anonymous voting when making collective decisions [53]. Several TB-affected members were also experienced advocates and community-health educators, which strengthened their ability to contribute confidently and influence deliberations.

In July 2023, Workshop 1 was held over two days in Ha Noi. On the first day, the workshop took place with the participation of 20 Leadership Group members, focusing on validating the categories generated from the Discover phase and identifying any gaps in the analysis. Participants were then asked to prioritize the challenges into opportunity areas. A ranked choice vote was held in which each Leadership Group member rated up to three opportunity areas for further exploration. To ensure inclusivity and balanced stakeholder engagement, the votes of each Leadership Group member, regardless of education, gender, age or qualification, were weighted equally and made anonymous. Based on the vote, the group agreed to advance five opportunity areas in the PCD process.

A structured facilitation process was conducted on the second day of Workshop 1 to co-develop an evaluation framework for ranking potential innovations in subsequent stages of the PCD process, consistent with participatory approaches that engage stakeholders in defining criteria and assessing priorities [54]. Participants worked in small groups to identify and discuss dimensions relevant to assessing TB innovations, which were collaboratively refined into an evaluation framework comprising three domains: fit and desirability, potential impact, and feasibility. Each domain was operationalized through the design prompts to support structured and transparent assessment across later phases (Table 5, presented in Results). Although feasibility was included as a core domain, participants agreed to defer its assessment until Workshop 3, when implementation planning and operational considerations would be addressed.

### Develop

A structured innovation mapping exercise (InnoScan) was conducted to identify existing product and service innovations with potential relevance for TB care delivery [55]. The scan reviewed innovations originating from low-, middle-, and high-income settings. Eligible innovations were required to be either specifically designed for TB or readily adaptable for use in TB care service delivery. Preference was given to digital or emerging technologies that were sufficiently mature for evaluation or real-world demonstration. Innovations requiring large-scale capital investment or duplicating existing initiatives were excluded. To contextualize the InnoScan to Viet Nam, the resulting list of innovations was mapped against opportunity areas identified in Workshop 1 by the study team. When multiple innovations addressed the same opportunity area, they were grouped into innovation categories.

The Leadership Group convened for a second workshop in September 2023, in Ho Chi Minh City with 19 participants to further prioritize innovation categories using the evaluation framework developed in Workshop 1. Early discussions indicated limited familiarity with the innovations identified during the mapping exercise. As a result, the workshop focused on building a shared understanding of the features of the proposed innovations before assessing the innovation categories against the evaluation framework. To support this, interactive displays were prepared for each of the 19 innovation categories. These included hands-on demonstrations, video materials, and live presentations by the study team, allowing workshop participants to engage directly with the proposed solutions. Following these interactions, the members scored each innovation category using a 5-point Likert scale across five evaluation questions drawn from the desirability and fit, and potential impact domains identified in Workshop 1. The questions included:

#### Desirability and Fit

1. To what level do you feel that this innovation category is/was relevant to you and your needs? (If a TB survivor, answer about your time on TB treatment)
2. To what level is the innovation category inclusive to everyone, across age groups, income levels, geography, and ethnicity?
3. How excited would you be to engage with, or benefit from this innovation category?

#### Potential Impact

4. How do you rate the potential of this innovation category to be implemented across multiple provinces in Viet Nam?
5. How likely do you feel this innovation is to solve the problems faced by the focus area in Viet Nam?

Data were submitted electronically through Google Forms, exported to Google Sheets, and then transferred to a pre-programmed analysis template in Microsoft Power BI (Version 2.149.1429.0), where average scores were calculated and visualized in real time in a matrix with Fit and Desirability on the x-axis and Potential for Impact on the y-axis. It was decided a priori that only three innovation categories would proceed, based on consensus.

In the weeks following Workshop 2, concept notes were developed by the study team for the three selected innovation categories to assess feasibility and define the scope and scale of potential interventions. While concept note development was researcher-led to ensure technical rigor and consistency across options, the framing was grounded in priorities identified by the Leadership Group in earlier phases. An indicative implementation envelope of approximately USD 1 million over up to two years informed the overall feasibility considerations, though this was not presented to participants as a fixed budget. Each concept note included a situation analysis, intervention description and goals, target populations, stakeholder roles and responsibilities, proposed innovations, expected outputs, timeline, and scale-up pathway. To ensure alignment with national priorities, feedback was first obtained from the NTP, and then from Leadership Group members during a 2-hour online consultation. All input was incorporated into the final concept notes, an example of which is provided in Additional File 3.

In a third workshop organized in late November 2023, in Hue City, 30 participants, with 16 from the Leadership Group, one representative from the Ministry of Health, five from Provincial Departments of Health, six from TB hospitals, and two international donors, reviewed the concept notes and engaged in structured discussions to provide perceptions and rationales for selecting one intervention over another for implementation. These discussions explicitly considered feasibility, including resource requirements, implementation complexity, and alignment with existing health system capacity and existing funding mechanisms, as outlined in the concept notes. Due to scheduling constraints, not all Leadership Group members were able to attend; however, all TB-affected members were present, and additional stakeholders were invited to ensure continuity of representation and decision-making capacity. The concept notes provided a structured basis for deliberation but did not predetermine the outcome. This composite stakeholder group conducted an anonymous vote determined by simple majority to select the intervention to be advanced for future evaluation and scale-up. An audio recording of the discussion was collected and a transcript was developed following the workshop.

## Results

### Discover

**Fig. 2:**
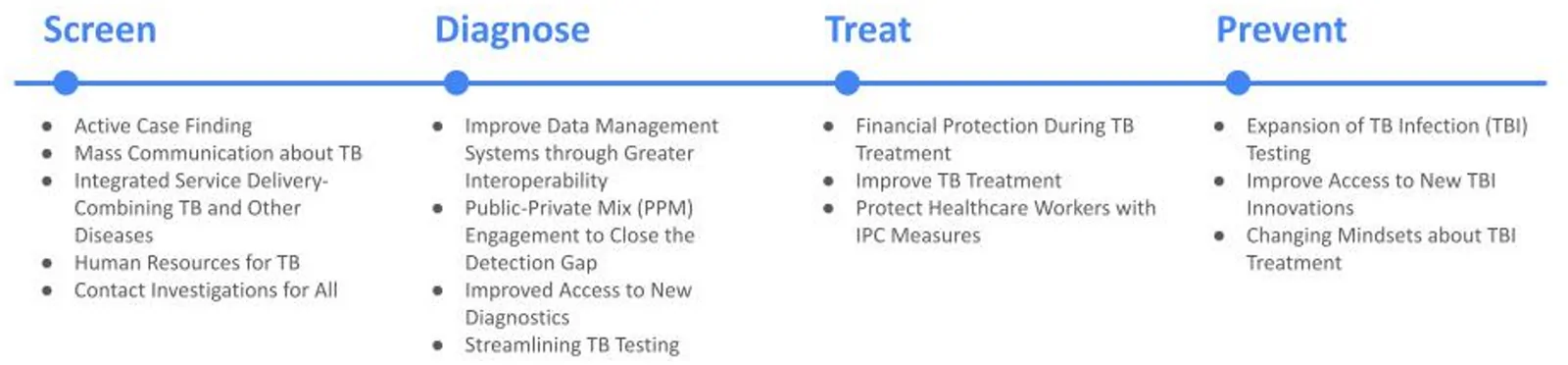
Categories of challenges in Viet Nam’s National TB Programme, grouped by the TB care cascade. Abbreviations: *IPC* Infection Prevention and Control, *PPM* Public-private Mix, *TB* Tuberculosis, *TBI* Tuberculosis Infection

Fifteen categories of challenges within Viet Nam’s TB program were mapped onto four predefined steps of the TB care cascade (Fig. 2). Active case finding (ACF) emerged as a central concern given the country’s gap between estimated TB incidence and notified cases, and interest in decentralizing diagnostic services to the commune level. Participants identified persistent challenges with limited human resources and insufficient community engagement. Contact investigation was consistently emphasized as critical for protecting families from TB transmission and enabling early detection. Diagnostic challenges were also a major concern, with participants calling for expanded access to molecular assays and improved sputum transport systems to reduce diagnostic delays. Stakeholders underscored inefficiencies in the coexistence of paper-based and electronic data management systems, pointing to the need for improved interoperability and deduplication of processes. Treatment-related priorities focused on improving treatment success through shorter regimens, greater access to fixed-dose combination drugs, and financial support mechanisms for underserved populations due to gaps in affordability and SHI coverage. Infection prevention and control (IPC) was raised as another challenge of importance, with healthcare staff reporting inadequate personal, administrative, and environmental protections. Finally, prevention challenges focused on TB infection (TBI) testing and treatment among high-risk groups, where limited access to new diagnostics and shorter regimens, along with healthcare worker perceptions, constrained uptake.

### Define

In the first consultation workshop, the Leadership Group reviewed, discussed, and validated the 15 categories of challenges. Input from members added nuance to four of the categories, leading to refinements in how these challenges were defined.

Following the discussion, the ranked choice voting selected: (1) *Active case finding* to expand diagnostic services, particularly in rural and underserved areas, and strengthen community engagement; (2) *Mass communication about TB* to reduce TB-related stigma, raise awareness, and encourage timely care-seeking; (3) *Improve data management systems through greater interoperability*, through integration of digital and paper-based systems to reduce administrative burdens and enhance efficiency; (4) *Human resources for TB* to address workforce constraints and improve service delivery; and (5) *Financial protection during TB treatment* to ensure equitable access to treatment by addressing cost barriers and improving affordability for underserved populations. Table 4 provides a summary of the ranked choice vote tally.

**Table 4:** Workshop 1 results prioritizing challenges in Viet Nam’s TB programme

Categories- Challenges identified in qualitative interviews	TB care cascade step	# of votes (*n* = 55)
Active case finding	Screen	14
Mass communication about TB	Screen	12
Improve data management systems through greater interoperability	Diagnose	6
Human resources for TB	Screen	5
Financial protection during TB treatment	Treat	4
Integrated service delivery - combining TB detection with other diseases	Screen	3
Improve TB treatment	Treat	3
Public-sector engagement to close the detection gap	Diagnose	2
Improved access to new diagnostics	Diagnose	1
Streamlining TB testing	Diagnose	1
Protect healthcare workers with IPC measures	Treat	1
Expansion of TB infection testing	Prevent	1
Improve access to new TBI innovations	Prevent	1
Changing mindsets about TBI treatment	Prevent	1
Contact investigations for all	Screen	0

*Abbreviations*: *IPC* Infection prevention and control, *TB* Tuberculosis, *TBI* Tuberculosis infection

Participants co-developed an evaluation framework consisting of 19 sub-dimensions, grouped into three overarching domains: fit and desirability, potential for impact, and feasibility. The framework was used to guide the prioritization of innovations in subsequent phases of the PCD process (Table 5).

**Table 5:** Evaluation framework to guide intervention selection, created by the Leadership Group

Dimension	Subdimensions	Guiding questions
Fit and Desirability	Innovation has desire / demand	Does the innovation stem from TB-affected people’s and healthcare providers’ needs?
	Ease of use	Is the innovation easy to use and understand? Is it more complex than existing solutions?
	National TB Programme	Does the innovation align with NTP priorities? Does the NTP see value and would support a scale-up?
	Privacy	Does the innovation keep user data private? Does it adhere to privacy regulations?
	Gender, Ethnicity, Culture	Is the innovation inclusive of all genders, ethnicities and age groups? Does the innovation align with Vietnamese culture and customs?
	Stigma	Do users face any stigma by using the innovation?
Potential for Impact	Health outcome	By how much does it improve health outcomes compared to the status quo? What are short-term and long-term benefits?
	Health economics	What is the benefit of the innovation in relation to its cost (cost-benefit analysis)?
	Education & Awareness	Does the solution increase education and awareness so that high-risk populations will access healthcare services?
	Opportunity area specific	To what degree does the innovation improve or aim to improve the challenges identified within the respective opportunity area?
	Equitable access	People: Does the innovation impact citizens, TB-affected people and healthcare providers? Does it impact all age and income groups? Who will not be able to access the solution? Why? Geography: Can the solution be implemented in the whole country and made accessible to all communities?
	Scope of solution	Does the solution support decentralized and integrated TB care? Can the solution be applied outside of the context of TB?
	Sustainability	Does the innovation have negative environmental consequences, and can it be operated sustainably?
	Scalability	Can the solution be scaled up in Viet Nam?
Feasibility	Cost	Is the price reasonable? Does the innovation incur additional costs to users? Healthcare system: Can the innovation be financed through existing sources of funding?
	Infrastructure requirements	What are the physical (e.g., temperature, humidity) and non-physical (e.g., digital interfaces, human resources) requirements?
	Workflow integration / interoperability	Does the innovation integrate within existing workflows and digital systems? How much time does it take to use the innovation? Does it speed up existing processes vs. create more work?
	Regulatory	Does the innovation comply with legal requirements? Is it allowed to be used widely?
	Procurement	Is the innovation commercially available? Are there tools / replacements in Viet Nam? Can the innovation be produced/manufactured/supplied in Viet Nam as opposed to being imported?

*Abbreviations: NTP* National TB Programme, *TB* Tuberculosis

### Develop

**Fig. 3:**
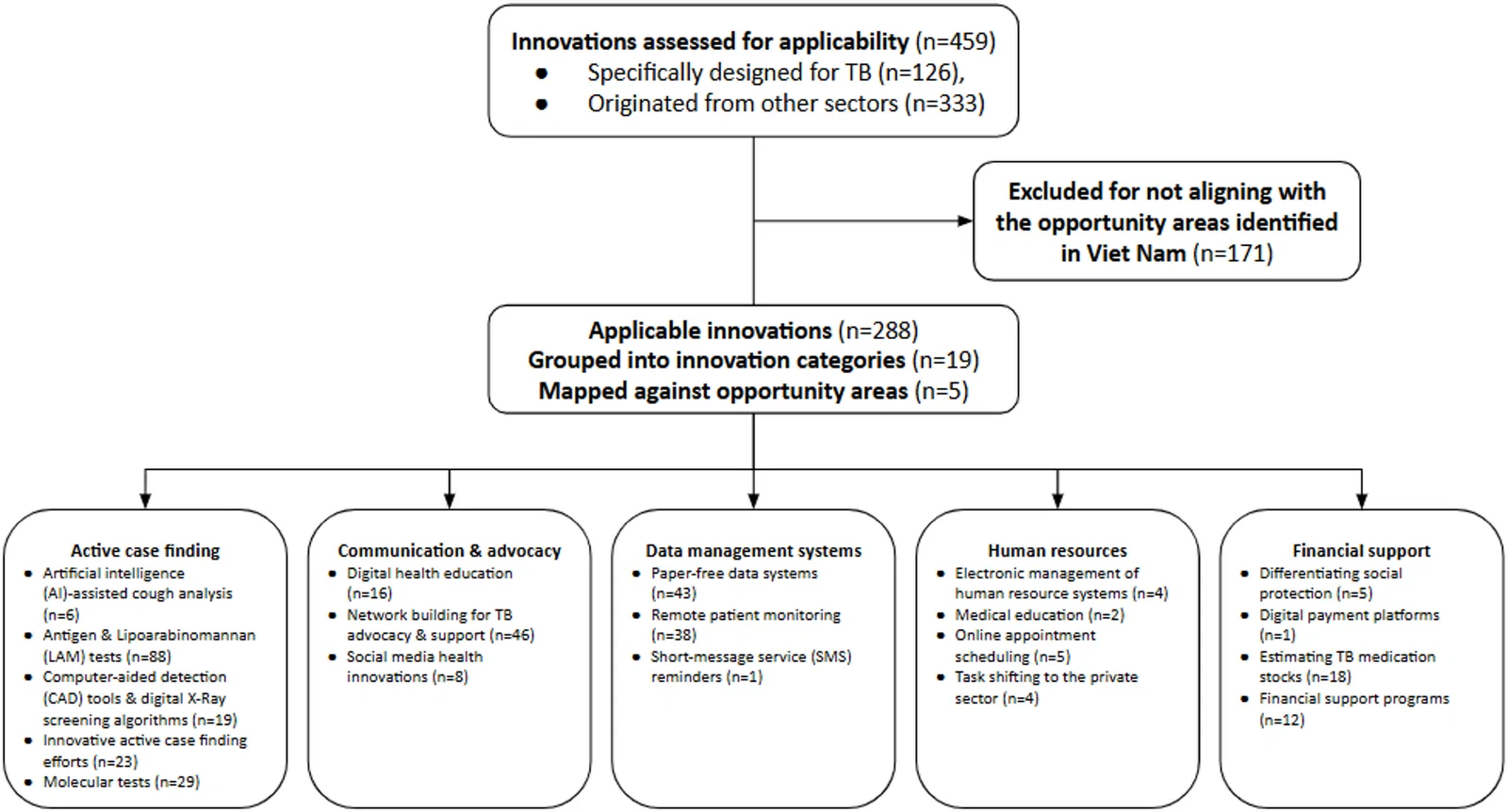
Identification, screening, and categorization of innovations assessed in the InnoScan

The InnoScan identified 459 total innovations. Among the applicable innovations, 126 innovations were specifically designed for TB, while 333 originated from other health or commercial sectors, but were adaptable for use in TB care service delivery. When assessed for the Vietnamese context, 171 did not align with one of the five opportunity areas identified in the Define stage and were excluded. These innovations were grouped into 19 categories and mapped against the five opportunity areas prioritized in Workshop 1. Figure 3 presents a summary of this process, while Additional File 4 details the materials presented for each innovation category in Workshop 2.

**Fig. 4:**
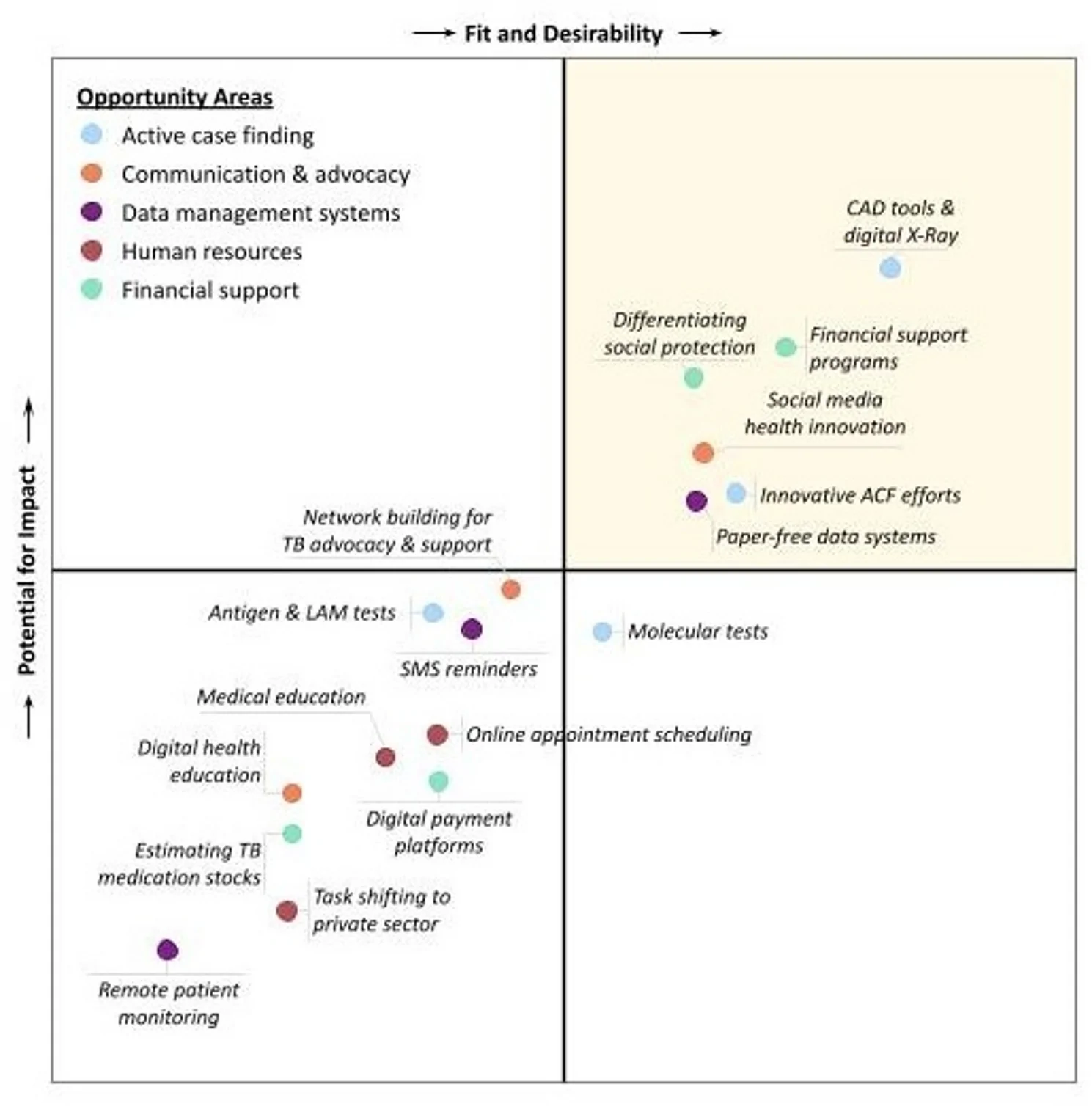
Evaluation matrix plotting innovation categories according to fit and desirability and potential for impact. Abbreviations: *ACF* Active Case Finding, *CAD* Computer-aided Detection, *LAM* Lipoarabinomannan, *SMS* Short-message Service, *TB* Tuberculosis

The second workshop generated comparative scores and visual outputs that supported structured prioritization of innovation categories. Assessment of the 19 innovation categories produced a ranked matrix that highlighted clear clustering patterns across domains of fit and desirability and potential impact (Fig. 4). The matrix displayed 17 innovation categories with comparable scores; two categories scored substantially lower and were not plotted. They included: artificial intelligence-assisted cough analysis and electronic management of human resource systems.

Six innovation categories were positioned in the upper-right quadrant, indicating high perceived fit and desirability and strong potential for impact. These included chest X-ray screening with computer-aided detection software, financial support programs, differentiated social protection measures, social media interventions, innovative approaches to active case finding, and paper-free data collection systems.

Scores varied across innovation categories within opportunity areas. Active case finding efforts ranked highest overall, whereas AI-assisted cough analysis was among the lowest-ranked categories. Financial support was the highest-scoring opportunity area, followed by active case finding, while human resources for TB consistently received the lowest scores.

During the workshop, a mismatch was observed between the innovation categories grouped under the human resources opportunity area and participants’ expressed needs. Participants consistently emphasized insufficient TB staffing to meet current workload demands, whereas the proposed innovations focused on management tools, training, appointment scheduling, and task shifting. Although these approaches were recognized as beneficial, they were not perceived to address workforce shortages. Human resource-related innovation categories consequently received consistently low scores.

Communication and advocacy were identified as cross-cutting elements relevant across intervention areas rather than as a standalone priority. In the discussion, two categories (financial support and differentiated social protection) were merged, since they both addressed challenges with the affordability of TB care. Thus, three innovation categories were selected for progression in the PCD process: (1) Integrated and localized computer-aided detection (CAD) software for reading CXR images (2) Paper-free data systems, and (3) Social and financial protection during TB care.

**Fig. 5:**
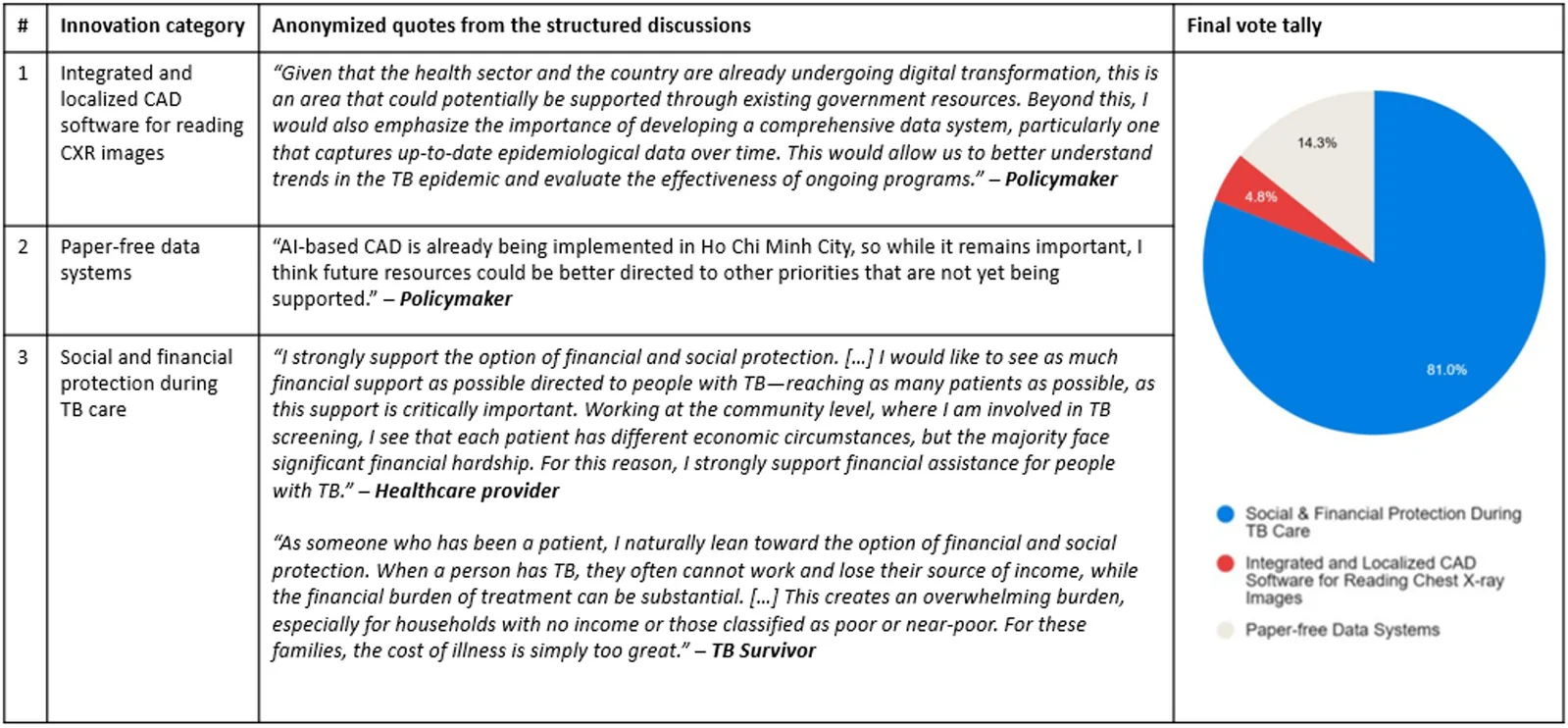
Final workshop results: prioritized innovation categories, illustrative participant quotations, and voting outcomes. Abbreviations: *CAD* Computer-aided Detection, *TB* Tuberculosis

In the third workshop, the concept notes assessing the feasibility of implementing the interventions in Viet Nam were discussed in detail. Participants raised concerns about the feasibility of several interventions. Participants noted that paper-free digital data systems would require substantial investment relative to the available budget and should be implemented at the level of the broader health system rather than as a TB-specific intervention. Similarly, while CAD software was viewed as a high-priority innovation, participants noted that it was already supported by external donors and therefore less appropriate for prioritization within this process. In contrast, participants emphasized the importance of addressing the financial burden of TB treatment on TB-affected people and households. Final voting results showed strong preference for social and financial protection (81.0%), followed by paper-free data systems (14.3%) and CAD software (4.8%) (Fig. 5).

## Discussion

This study applied a structured PCD process to enable TB-affected people and a diverse range of health system stakeholders to jointly prioritize future TB investments in Viet Nam, demonstrating that participatory co-design can meaningfully complement national planning processes in a centralized, hierarchical health system where community engagement has historically been limited. Through iterative qualitative inquiry and deliberative workshops, participants identified key constraints across the TB care cascade, co-developed an evaluation framework grounded in lived experience, and selected social and financial protection as the top investment priority. This outcome clearly reflects community-defined needs rather than solely technical or epidemiological criteria. This progression moves beyond the consultative engagement that characterizes many health planning processes, advancing along the WHO continuum of community engagement toward collaboration and shared decision-making, though stopping short of full empowerment given the researcher-led framing of options presented for final selection [21]. In doing so, the study contributes to a growing body of evidence that qualitative and participatory design approaches can improve the relevance and responsiveness of health policies and interventions, particularly when TB-affected people are engaged at early stages of problem identification and prioritization rather than consulted after priorities have already been set [9, 56–58].

Findings from the Discover and Define phases largely reinforced priorities identified in the previous NSP 2021–2025, including gaps in access to diagnosis, treatment continuity, and the financial burden of TB care [59]. The added value lay not in identifying entirely new challenges but in reframing existing priorities through lived experience, revealing misalignments between system-level strategies and the day-to-day realities of TB-affected people and frontline providers. This reframing is itself a meaningful contribution, highlighting how well-documented problems are actually experienced in practice, and where conventional planning processes may systematically undervalue community perspectives.

A distinctive feature of the Develop phase of the PCD process in Viet Nam was the inclusion of an explicit learning component during Workshop 2, in which participants engaged directly with proposed product and service innovations through interactive demonstrations. This shared exposure helped establish a common baseline of understanding and supported more informed and equitable deliberation. While the previous NSP development process in Viet Nam employed a structured, evidence-driven approach to prioritization, including patient pathway analysis and modelling to identify a package of high-impact interventions, it did not explicitly articulate a transparent process for comparative ranking of individual interventions against predefined criteria [59]. While WHO guidance on NSP development emphasizes multisectoral engagement and stakeholder participation, it similarly does not explicitly incorporate structured learning or capacity-building components within prioritization processes [31]. By equipping participants, including those with limited prior exposure to global TB innovations, with the knowledge required to assess alternatives, the Develop phase, and specifically Workshop 2, strengthened the quality and inclusiveness of prioritization.

The Develop phase marked a transition from consultative engagement to stakeholder-led decision-making. Unlike many health planning processes, where stakeholder input is sought but decision-making authority remains centralized, the Leadership Group was responsible for selecting the intervention for future investment. This distinction between consultation and deliberative decision-making is well recognized in the participatory governance literature [60–62]. While earlier phases were consultative, the Develop phase operationalized priorities through a structured decision-making process that positioned the Leadership Group, alongside additional stakeholders, as final decision-makers. However, decision-making authority over final selection should be distinguished from the framing of options available for selection, as concept notes were developed by the study team, meaning that the boundaries of the decision space were researcher-defined, even as the final choice remained with stakeholders [63]. This hybrid model reflects a pragmatic balance between technical rigor and participatory governance, and is worth acknowledging as a design feature rather than a limitation alone.

Several methodological limitations should be considered. First, the study relied on qualitative data and participatory synthesis, which involved interpretive steps in coding, categorization, and the development of design prompts that may have introduced researcher bias despite team-based analysis and validation processes. Second, the composition of the Leadership Group, while intentionally diverse, was not proportionally balanced across key characteristics, such as gender, geographic representation, or stakeholder type, which may have influenced deliberations and final prioritization. Third, participation of the Leadership Group varied across phases, which may have affected the continuity of perspectives informing the iterative design process. Fourth, the development of concept notes by the study team, rather than through a co-design process with the Leadership Group, introduced researcher influence over the framing of options available for final selection. While grounded in priorities identified by stakeholders in earlier phases, this approach meant that the boundaries of the decision space were partially researcher-defined, which should be considered when interpreting the degree to which final priorities fully reflect community-led decision-making. Finally, the study did not incorporate formal economic evaluation or quantitative prioritization methods (such as the analytical hierarchy process), limiting the ability to compare interventions based on cost-effectiveness or to generalize findings beyond the study context.

Overall, the process was resource-intensive and time-consuming, spanning ten months, although such constraints are common in participatory research [64, 65]. The approach integrated multiple interconnected qualitative, participatory, and deliberative methods across successive phases of the Double Diamond framework, requiring substantial expertise in qualitative research, facilitation, and participatory design, and the TB care ecosystem, as well as a multidisciplinary team with experience across the full range of TB service delivery. Delivery depended on multiple senior implementers, increasing costs and potentially limiting the transferability of the approach to settings or organizations with less technical capacity. Nonetheless, the process, decision points, and facilitation methods are described in detail in the methods, allowing others to judge which elements are essential and which can be tailored to local resources and context. Consistent with participatory approaches, we anticipate that future users would adapt the process to their context while preserving its core principles of co-production, deliberation, and community leadership. Engagement was also shaped by contextual factors, including competing professional responsibilities and varying familiarity with participatory methods and innovation concepts, which influenced who was able to engage consistently and how actively participants contributed across stages.

Managing the influence of more powerful, senior or technically experienced stakeholders in the Leadership Group also posed difficulties, as they risked dominating discussions and expanding project scope. Deliberate facilitation strategies were utilized to mitigate this, including equal voting rights, anonymous decision-making, and early input from TB-affected participants, strategies widely recommended in participatory and design-based research [60, 66, 67].

Lessons from this study suggest several implications for future co-design and intervention development processes. Applying PCD earlier in planning cycles may help align investments with lived experience before priorities become fixed through technical or funding processes. Future applications may be made more efficient by leveraging established toolkits, pre-prepared facilitation materials, and evidence from analogous settings, rather than rebuilding process elements that have already been tested [68]. Utilizing existing data and ideas from analogous settings may expedite exploratory design stages, while rapid evidence assessments and focused stakeholder dialogues may also enhance efficiency. Incorporating digital tools, such as virtual focus groups, online surveys, and automated coding or visualization platforms, offers potential to reduce logistical burdens and widen participation [69]. Local capacity development, such as educating community members and local staff in facilitation and qualitative research, has the potential to lessen dependence on experts while also promoting sustainability [70]. Consistent with participatory research literature, strategies to reduce power imbalances, such as structured facilitation, training on equitable engagement, and the use of anonymous voting or ranking methods, remain critical to ensuring equitable participation [71].

The transferability of this PCD approach lies in its structured yet adaptable design, allowing it to be applied across settings while tailoring stakeholders, tools, and implementation to local contexts [9, 72]. This was reflected in its concurrent application in Uganda, where the same PCD process yielded different priority interventions, underscoring how outcomes are shaped by local context while the underlying approach remains consistent [68]. However, as with other participatory approaches, outcomes are inherently shaped by the composition of participants and local context, underscoring the importance of reflexive practice and transparent reporting in assessing the applicability of findings across settings [73].

The selection of social and financial protection reflects both the lived economic challenges faced by TB-affected households and evolving policy shifts in Viet Nam. Despite recent reforms in social health insurance and efforts to expand domestic financing, financial hardship remains a major barrier to care, and existing mechanisms, including the Patient Support Foundation to End TB (PASTB), have struggled to meet demand [74–76]. Evidence supports social protection interventions, including conditional cash transfers, food support, and transportation subsidies, as effective in reducing barriers to care and improving treatment adherence [77–79]. Global strategies, including the End TB Strategy and the Resolution passed at the 2023 United Nations High-Level Meeting on TB, emphasize reducing catastrophic costs and expanding social protection [2, 80]. However, these areas have not consistently been prioritized by major donors, and recent reprioritization under the Global Fund Grant Cycle 7 has further restricted support for interventions, such as nutritional assistance to high-need groups, reflecting broader funding constraints affecting integrated support for TB-affected households [81]. These trends are expected to intensify under Grant Cycle 8, alongside wider reductions in global health financing and an increasing emphasis on narrowly defined “life-saving” interventions [82–84]. In this context, the prioritization of social and financial protection highlights the value of participatory processes in identifying locally relevant investment priorities that may otherwise be underemphasized in funding decisions.

## Supplementary Information


Supplementary Material 1.



Supplementary Material 2.



Supplementary Material 3.



Supplementary Material 4.


## Data Availability

The datasets generated and/or analyzed during the current study are available from the corresponding author upon reasonable request, subject to confidentiality and ethical restrictions to protect the privacy of qualitative interview participants.

## Abbreviations

CAD: Computer-aided Detection
CSOs: Civil Society Organizations
HCD: Human-Centered Design
IPC: Infection Prevention and Control
IRB: Institutional Review Board
LMICs: Low- and Middle-Income Countries
NGOs: Non-governmental Organizations
NSP: National Strategic Plan
NTP: National Tuberculosis Program
PCD: People-Centered Design
RTC: Re-imagining TB Care
SHI: Social Health Insurance
TB: Tuberculosis
TBI: Tuberculosis infection
WHO: World Health Organization
