# Supplementary material for: Engaging tuberculosis-affected people in investment decisions: applying a people-centered design approach in Viet Nam
Source: BMC Public Health. 2026 Aug 27;26:2466. doi: 10.1186/s12889-026-29213-z (PMC13523514; doi:10.1186/s12889-026-29213-z)
Supplement: Supplementary file 1 [file 12889_2026_29213_MOESM3_ESM.pdf]

CONCEPT NOTE

Integrated and Localized CAD Software for Reading Chest X-ray Images

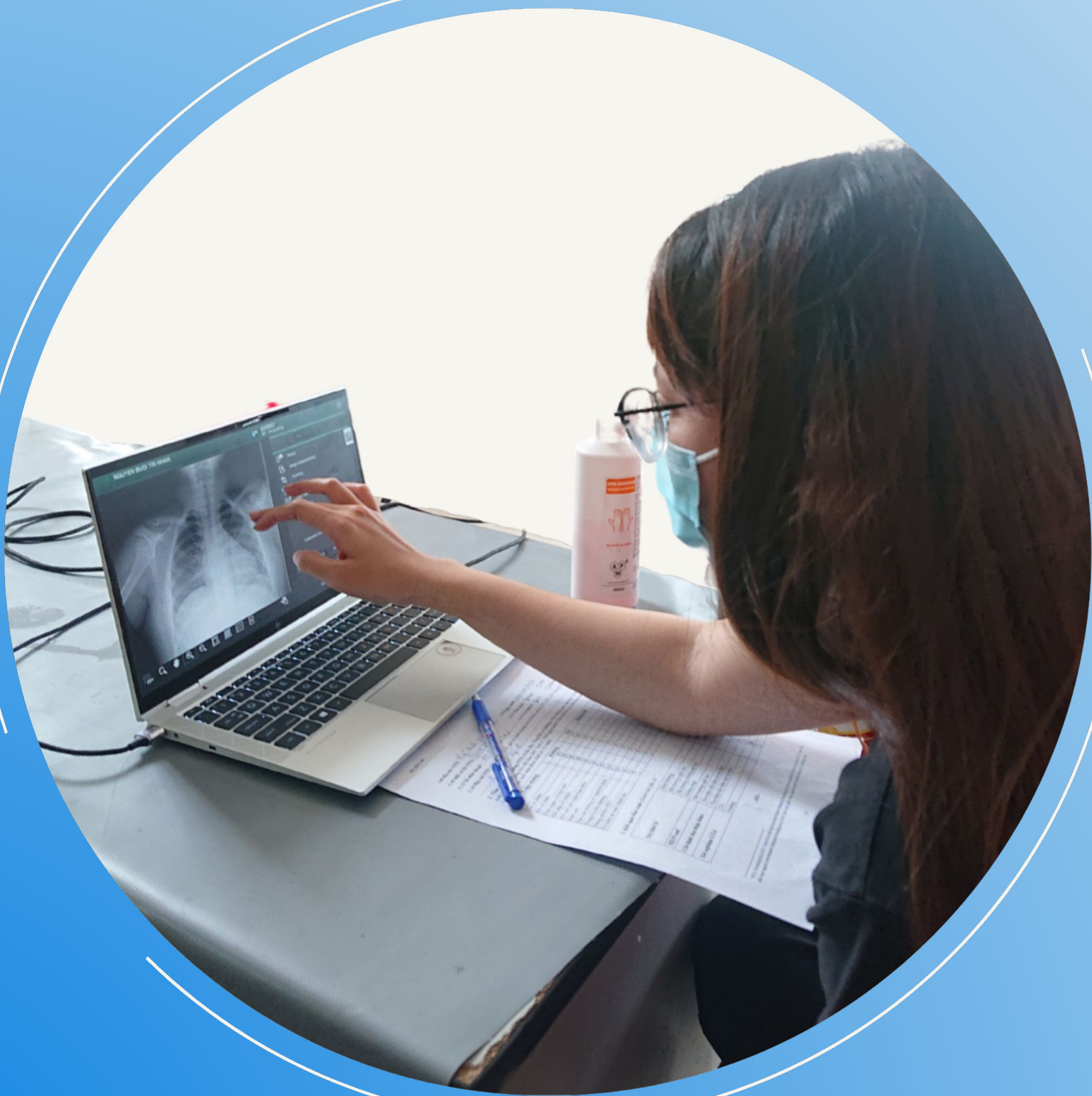

Photo: FIT staff using CAD software to read chest X-ray films

Challenges and The Opportunity Areas

- An estimated 35% of people with TB in Viet Nam's last TB prevalence survey[1] did not report TB symptoms (such as cough) and were only indicated for sputum testing and diagnosed with TB through screening with a chest X-ray
- However, the quality and accuracy of chest X-ray interpretation by radiologists can vary greatly across settings; it is possible for signs of TB on an X-ray to be interpreted as an abnormality not associated with TB or to be missed entirely
- CAD software can read and interpret chest X-ray images in a standardized and customizable fashion
    - Independent evaluations, including one conducted by the NLH and FIT in Viet Nam[2], show that CAD software perform at least as well as radiologists
    - The World Health Organization now recommends using CAD software for chest X-ray interpretation during TB screening[3], but only as radiologist replacement
- Very few studies have investigated how CAD software can be used as a diagnostic aid for radiologists to ensure the accuracy and quality of their interpretation
    - FIT piloted a CAD software deployment in one public-private mix (PPM) clinic in Ho Chi Minh City (HCMC) between June 2022 and 2023
    - During the pilot the CAD software increased TB detection by +44%[4] compared to the radiologist alone

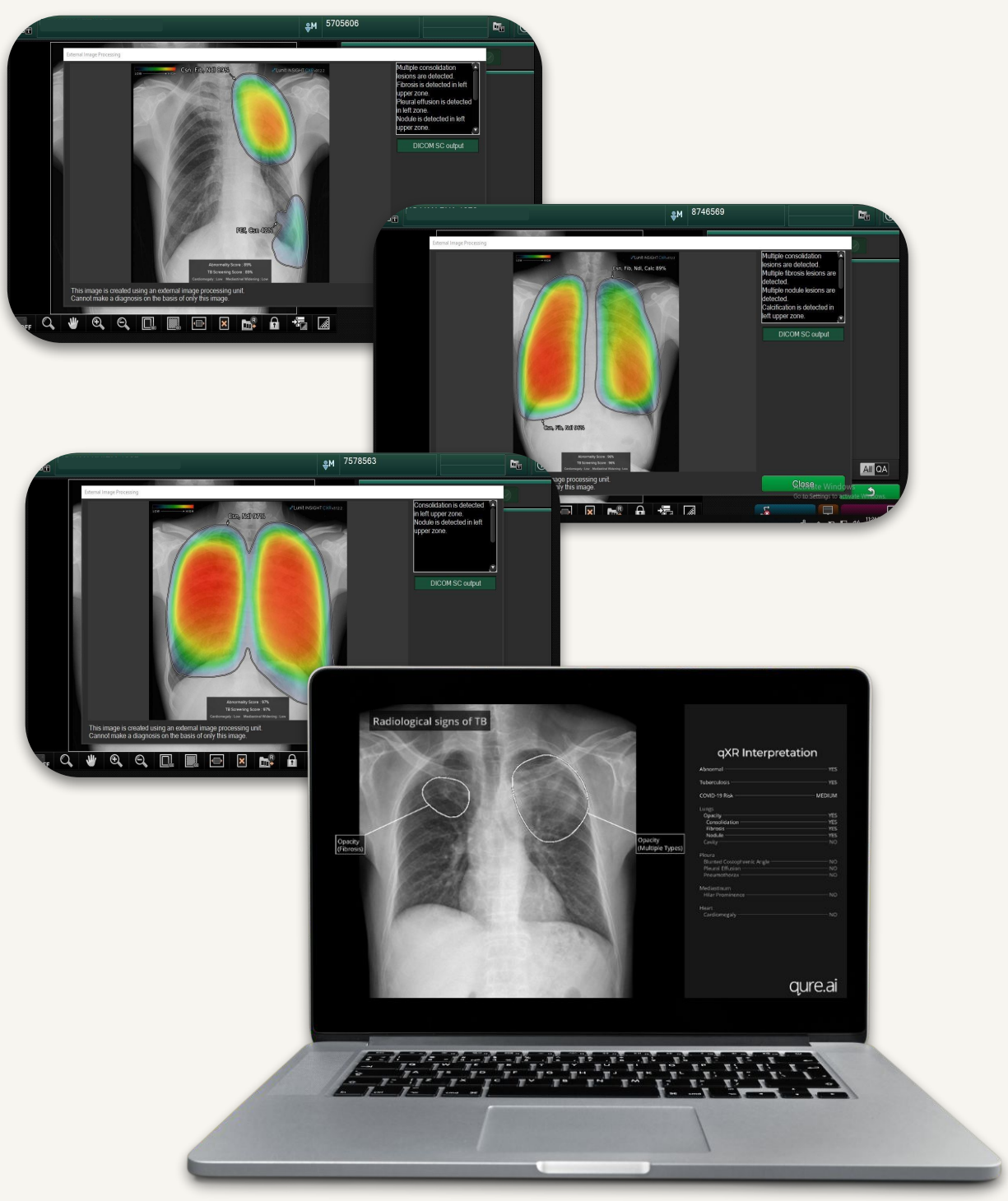

Photo: Diagnosis of chest X-rays on CAD software

1.Huong Van Nguyen, Edine Tiemersma, Hoa B Nguyen, Frank Cobelens, Alyssa Finlay, Philippe Glaziou, Cu Huy Dao, et al. 2020. *The Second National Tuberculosis Prevalence Survey in Vietnam.* https://doi.org/10.1371/journal.pone.0232142.
2.Andrew James Codlin, Thang Phuoc Dao, Luan, Rachel Jeanette Forse, Vinh Van Truong, Ha Dang, Lan Huu Nguyen, et al. 2021. *Independent Evaluation of 12 Artificial Intelligence Solutions for the Detection of Tuberculosis*. https://doi.org/10.1038/s41598-021-03265-0.
3.WHO. 2021. *WHO consolidated guidelines on tuberculosis: Module 2: screening – systematic screening for tuberculosis disease*. https://www.ncbi.nlm.nih.gov/books/NBK569341/#__NBK569341_dtls__.
4.AJ Codlin, TP Dao, DT Le, HB Huynh, LNQ Vo, R Forse, K Sidney Annerstedt, J Lundin, K Lönnroth, TV Ngoc, HTM Dang, & LH Nguyen. 2023. *The added value of CAD software for external quality assessment (EQA) during TB screening at a private clinic in Viet Nam*. https://docs.google.com/presentation/d/1TdEAqPLxnoT_enyFH7yn3tFekOpjO77i/edit?usp=sharing&ouid=116485665208862985304&rtpof=true&sd=true

Intervention Description

People visiting collaborating PPM facilities will first be registered and clinically assessed for the presence of TB symptoms using a custom-built mHealth app. They will then be screened using the facility's X-ray system, regardless of their symptom presentation. Chest X-ray images will be immediately interpreted by an on-site radiologist, and in parallel by VinBrain's DrAid CAD software[1]. The on-site radiologist and CAD software will interpret the chest X-ray images in parallel, blinded to the other's results. Participants with an abnormal result from either the radiologist and/or the CAD software (even when they disagree with each other) will be requested to provide a sputum specimen for diagnostic testing using the Xpert MTB/RIF Ultra assay.

This work will be nested within FIT's existing PPM Model 5 program, which is funded by the Global Fund[2]. FIT will work closely with the RTC's Leadership Group to co-select 1-3 PPM facilities per province across 15 provinces (see list below), comprising 40% of Viet Nam's total population, where existing TB screening activities will be enhanced by the incorporation of the CAD software. We believe that over 1,000,000 people will be screened by chest X-ray as part of this work, and that there are substantial opportunities to detect TB missed by the radiologist through standardized reading of the X-ray images by the CAD software. In addition, we will explore opportunities to use the CAD software to interpret X-ray images for diseases other than TB and to generate standardized radiology reports to support a practical approach to lung health. PCD principles will be applied in acceptability/desirability, feasibility, and effectiveness assessments during the intervention implementation period.

Intervention Co-design

We will conduct the user experience assessments and will then work with VinBrain to improve CAD software interfaces to make it more user-friendly.

1.VinBrain. https://vinbrain.net/
2.The Global Fund. October 2022. *The TB Quarterly Update: Innovative Approaches to Finding and Treating Missing People with TB.* https://www.theglobalfund.org/media/12452/tb_2022-10-quarterly-tuberculosis_update_en.pdf.

Scope & Scale

1-3 PPM facilities per province in 15 provinces where the Global Fund's PPM Model 5 project is operating and FIT has implementation approvals. These provinces include:

NORTHERN	CENTRAL	SOUTHERN
Ha Noi	Da Nang	Ho Chi Minh City
Hai Phong	Thua Thien Hue	Can Tho
Ha Giang	Binh Dinh	Binh Duong
Thanh Hoa	Quang Nam	Ba Ria - Vung Tau
Thai Nguyen		Long An
		Vinh Long

Roles & Responsibilities

PPM Facility Staff

- Collect sputum specimens for Xpert testing based on the CAD software result, even when it disagrees with the on-site radiologist
- Maintain and troubleshoot the CAD software deployment in collaboration with FIT
- Participate in PCD process on how to optimize the CAD software deployment

NLH & PLH Staff

- Develop SOPs and training materials on CAD software deployment
- Train PPM providers
- Participate in the PCD and evaluation processes
- Issue policy update on CAD software use after evidence generated and synthesized
- Results dissemination

FIT Staff

- Install CAD software at PPM health facilities
- Maintain and troubleshoot the CAD software deployment in collaboration with PPM health facilities
- Evaluate the feasibility and effectiveness of the CAD software deployment
- Use PCD principles to optimize the CAD software deployment
- Results dissemination

Goals

- Improve the capacity of private sector health facilities to do TB screening
- Detect more TB in PPM facilities
- Determine the real-world implementation feasibility and added value of using CAD software to support chest X-ray interpretation in conjunction with a radiologist
- Establish policy on CAD software use in Viet Nam and possibly inform a revision of WHO policy to include more ways in which CAD software can be used

Target populations served

- Healthcare-seeking people visiting PPM facilities
- Individuals whose CXR abnormalities are missed by the PPM radiologist
- PPM facilities - capacity and reputation building

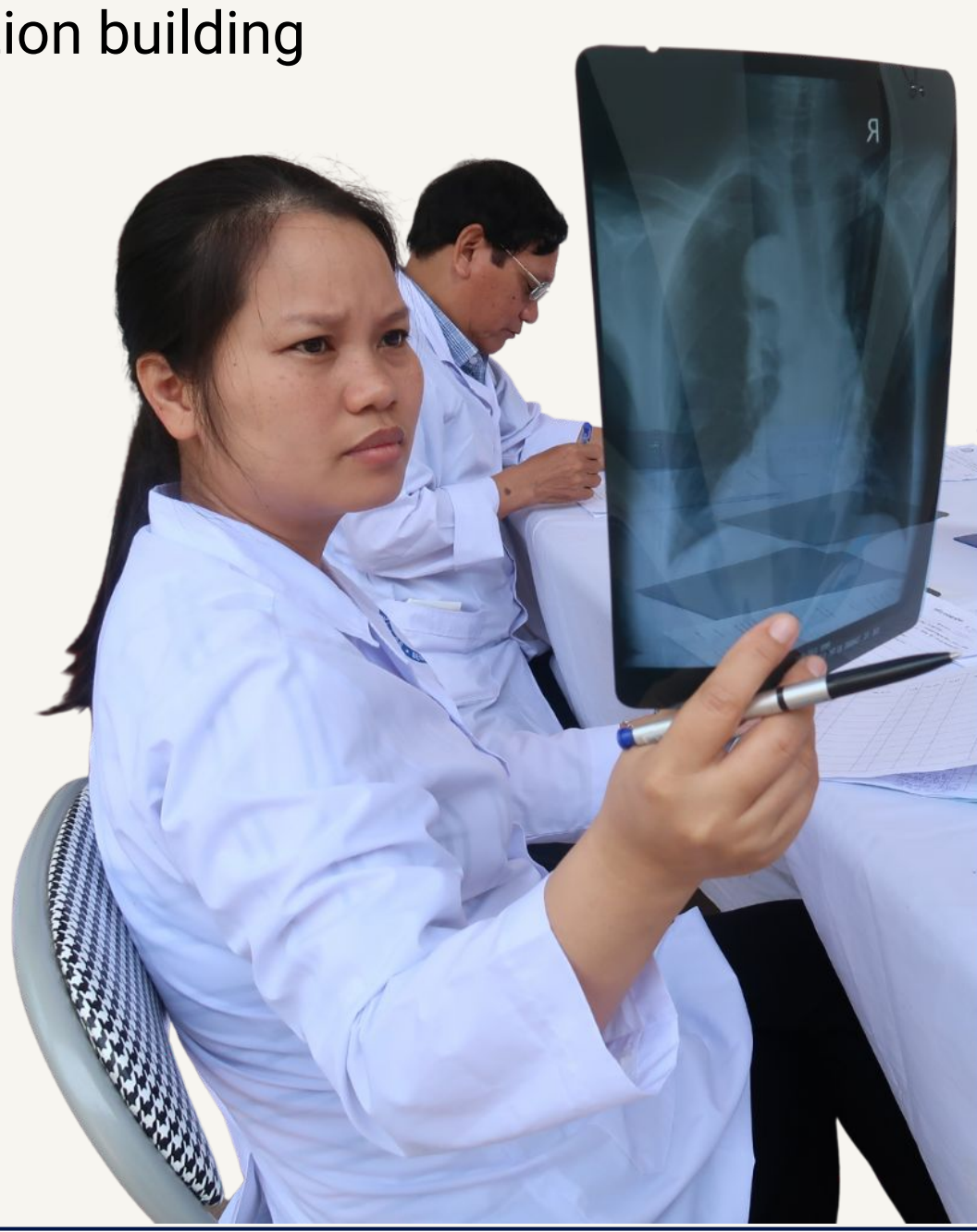

Photo: An X-ray film is being interpreted by a radiologist

Potential product and/or service delivery innovation

- Intensified TB case finding reaching more than 1 million people
- Optimization of VinBrain's DrAid CAD software deployment

Potential country- and global-level partners

**CAD service provider**	VinBrain
**Radiography system providers**	Fujifilm, Konica Minolta, DRTech, Canon, Siemens, General Electric
**Health Services**	National Reference Lab, Provincial Lung Hospitals / Provincial TB Programs, Provincial Respiratory Disease Associations, and private providers

Uniqueness of proposed project in the donor landscape

- Approach VinBrain to donate software licenses (or to offer concessional pricing); Global Fund can support PPM staff and incentives to support sputum collection and testing
- Donors are not yet supporting deployments of any Vietnamese CAD software utilizing the extensive PPM network. However, USAID and Global Fund are supporting community-based active TB case finding campaigns and the evaluation of VinBrain and Qure.ai CAD softwares
- The evidence gained by this intervention could inform Vietnamese policy updates related to use of CAD in PPM facilities; it would be paired with evidence from community-based campaigns
- Very few implementers are using CAD as a diagnostic aid for a radiologist, rather than as an outright radiologist replacement; Evidence from Viet Nam may inform WHO policy revision on CAD software

Introduction & Scale-up Pathway

PRE-APPROVAL

- Obtain approvals under Decree 80 (The Vietnamese law that governs all foreign donations to non-profit organizations such as FIT)
- Negotiate procurement (or donation) of VinBrain software licenses
- Custom installation of CAD software into X-ray systems at PPM health facilities

LAUNCH

- Sign contracts and initiate screening across the 15 provinces that are part of the Global Fund's PPM Model 5 program
- Train facilities on how to integrate CAD software into their diagnostic flows
- Screen over 1,000,000 participants with chest X-ray and DrAid
- Continuously evaluate the feasibility and effectiveness of CAD-supported TB screening via a PCD process in order to optimize this activity

SCALE-UP

- NLH/NTP to issue circular on the use of CAD software to support TB screening; RTC will provide all evidence related to facility-based usage of CAD software while other donors will fund community-based guidance
- Assessments will be conducted using PCD principles to understand acceptability, desirability, feasibility, and effectiveness; modifications to implementation plans will be made based on findings
- Continue CAD software-supported TB detection within the PPM Model 5 project by the transition of the strategy to the Global Fund's 2024-26 cycle

Research and policy outputs

- Scientific publication of CAD software feasibility and added value during TB screening at PPM health facilities
- Policy dialogue on CAD software use to support chest X-ray screening for TB
- Published guidance on facility-based CAD software use in Viet Nam

Potential key success indicators

- Adoption and utilization of CAD software to indicate sputum testing [feasibility]
- Incremental / additional people diagnosed with TB due to the CAD software (above what the radiologist indicated) [effectiveness]
- Issuance of national guidance on CAD software use during TB screening [scalability]

Potential timeline

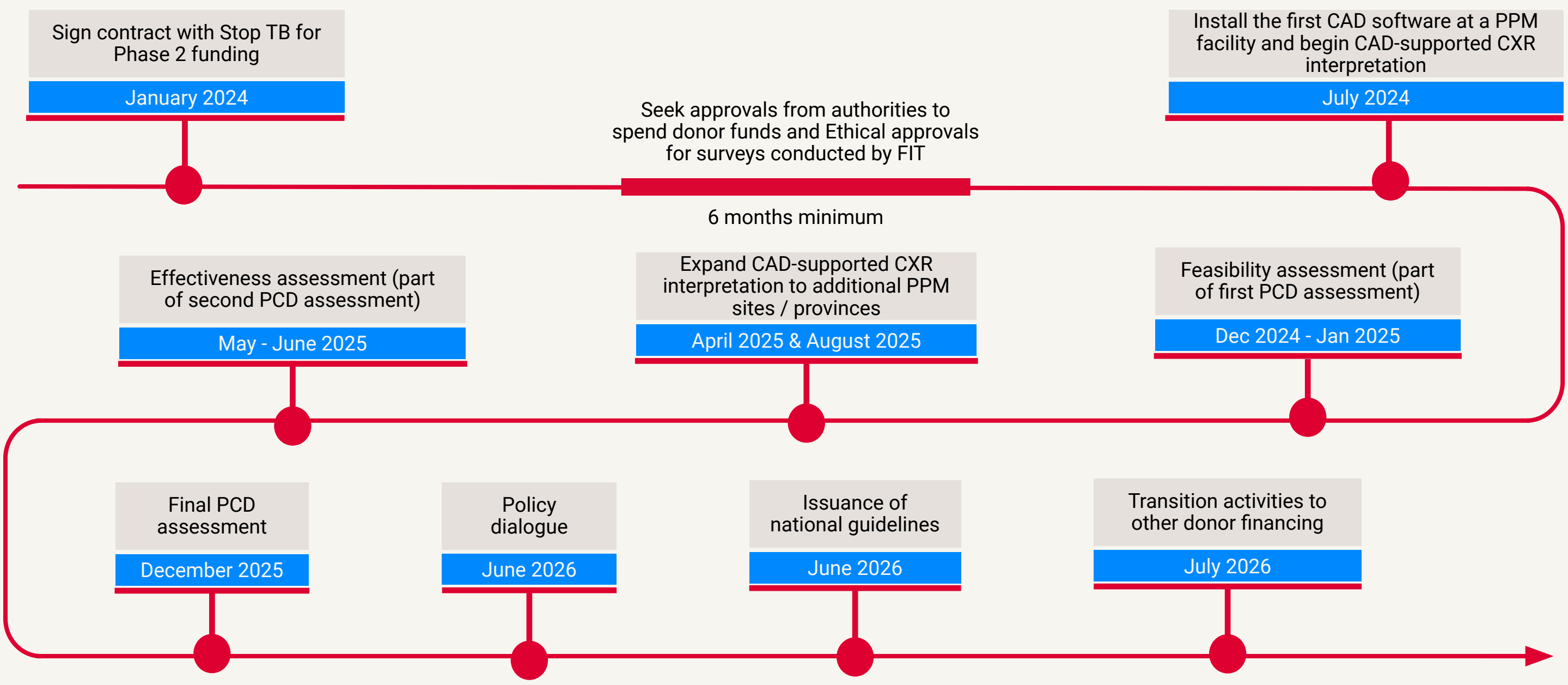

FRIENDS FOR INTERNATIONAL TUBERCULOSIS RELIEF

KOICA Korea International Cooperation Agency

Global Disease Eradication Fund KOREA

Stop TB Partnership hosted by UNOPS

RE-IMAGINING TB CARE
