# Supplementary material for: Engaging tuberculosis-affected people in investment decisions: applying a people-centered design approach in Viet Nam
Source: BMC Public Health. 2026 Aug 27;26:2466. doi: 10.1186/s12889-026-29213-z (PMC13523514; doi:10.1186/s12889-026-29213-z)
Supplement: Supplementary file 2 [file 12889_2026_29213_MOESM1_ESM.docx]

| **Section and topic** | **Item** | **Reported on page No** |
| --- | --- | --- |
| 1: Aim | Report the aim of PPI in the study | 5-6 |
| 2: Methods | Provide a clear description of the methods used for PPI in the study | 6-15 |
| 3: Study results | Outcomes—Report the results of PPI in the study, including both positive and negative outcomes | 15-22 |
| 4: Discussion and conclusions | Outcomes—Comment on the extent to which PPI influenced the study overall. Describe positive and negative effects | 23 |
| 5: Reflections/critical perspective | Comment critically on the study, reflecting on the things that went well and those that did not, so others can learn from this experience | 23-27 |
