# Supplementary material for: Engaging tuberculosis-affected people in investment decisions: applying a people-centered design approach in Viet Nam
Source: BMC Public Health. 2026 Aug 27;26:2466. doi: 10.1186/s12889-026-29213-z (PMC13523514; doi:10.1186/s12889-026-29213-z)
Supplement: Supplementary file 3 [file 12889_2026_29213_MOESM2_ESM.docx]

# Supplementary File 1. Interview Guides for the Discovery Stage

## For TB-affected individuals/ TB community

1. Intro question/icebreaker: demographic information
2. What worked well during your TB care?
   2.1. Access: travel, cost, location of provider/facility, diagnostic technology, Social health insurance (SHI)
   2.2. Convenience: time required, booking appointment/waiting at clinic, clinic hours, proximity of diagnosis to home/work
   2.3. Support: empathy, education, social support
   2.4. Acceptance: trust, stigma
3. Describe the challenges you faced during your TB journey?
   3.1. Probe for diagnosis
   3.2. Probe for treatment
   3.3. Probe for follow-up
   3.4. Prompt for greatest challenge
4. From your experience, what l was the largest area for improvement in your TB care journey? / What would you like to change to make TB care better?
   4.1. Probe for Access, Convenience, Support & Acceptance
5. Let us imagine a world, where we could completely redesign the TB care journey. What would you change?
6. What are your impressions of TB products/innovations?

## For Policymakers

1. Intro question/icebreaker
2. We have reviewed the National Strategic Plan which covers 2021-2025 for the TB program in Viet Nam. We are halfway through implementing this plan. What do you feel should be the main priority areas for improvements in TB services in the country?
   - Add NSP priorities
   - What outside of the NSP should be added?
3. From these priorities, what do you personally feel is most important?
4. How do you recommend that we go about making these changes?
5. From your knowledge of TB innovations / products / supportive systems that are available in other countries, what is Viet Nam lacking?
6. What do you feel is working well within the TB program?
   - Probe for Access, Convenience, Support & Acceptance
7. What are the problems in the TB cascade of care?
   - Probe for Access, Convenience, Support & Acceptance
8. What lessons should TB learn and/or adopt from the innovations / products / supportive systems that were made available during the COVID-19 pandemic?
9. What are your impressions of TB products/innovations?
10. What would it take to test and roll-out these products/innovation nation-wide?

## Healthcare providers, NGOs & CSOs & private sector providers

1. Intro question/icebreaker: Can you tell me your experience with TB care?
2. What do you feel is working well within the TB program?
   a. Probe for Access, Convenience, Support & Acceptance
3. What are the problems in the TB cascade of care?
   a. Probe for Access, Convenience, Support & Acceptance
4. Describe what you know of the TB program’s outreach to vulnerable communities.
   a. How effectively are we reaching these different groups of people?
   b. What could be done better to address their specific needs?
5. What services in the TB care cascade do you think should be prioritized for improvement?
   a. National-level
   b. Provincial-level
   c. District/commune- level
6. How do you recommend that the government go about making these changes?
7. What lessons should TB learn and/or adopt from the innovations / products / supportive systems that were made available during the COVID-19 pandemic?
8. What are your impressions of TB products/innovations?
