# Supplementary material for: Engaging tuberculosis-affected people in investment decisions: applying a people-centered design approach in Viet Nam
Source: BMC Public Health. 2026 Aug 27;26:2466. doi: 10.1186/s12889-026-29213-z (PMC13523514; doi:10.1186/s12889-026-29213-z)
Supplement: Supplementary file 4 [file 12889_2026_29213_MOESM4_ESM.docx]

# Supplementary File 4. Materials Presented for 19 Innovation Categories at Workshop 2

| **Innovation category #.** | **Opportunity area** | **Category** | **Description** | **Examples presented in workshop** | **Presentation method** |
| --- | --- | --- | --- | --- | --- |
| 1 | Communication & Advocacy | Social media health innovation | Custom, national-level awareness campaigns. Using existing / concluded campaigns as best practice examples | Social media platforms: YouTube/Google Health, TikTok, Zalo, Facebook.  Existing awareness campaigns: TOSS-TB, If you love me, Check am O, Project Axshya - BuLeadership Groupam Bhai | TV displaying slideshow of existing awareness campaigns |
| 2 | Communication & Advocacy | Network building for TB advocacy and support | Existing programs that try to involve the community in TB education and awareness | TB champion clubs formed and connected online; Champions conduct outreach | TV displaying slideshow of existing community programs- Jannis |
| 3 | Communication & Advocacy | Digital health education | Applications / websites etc. that offer educational service for people with DS- and DR-TB, and their contacts. | V-SMART MDR-TB App | TV displaying video of app instruction:<https://www.youtube.com/watch?v=1j0GT_-bS2g> |
| 4 | Data management systems | SMS Reminders | Applications designed to collect data, e.g., names, socioeconomic data, symptoms etc. They can allow frontline healthcare workers to register people and sync the data into national databases. They allow for automated messaging of people with TB about their treatment and follow-up. | TB ACIS 365 | TV displaying demo on TB ACIS 365 |
| 5 | Data management systems | Health data management platform- Paper-free TB data systems | Platforms designed for large-scale management of healthcare data. These systems can allow for full transition away from paper-based reporting. | VITIMES | TV displaying demo video on VITIMES |
| 6 | Data management systems | Remote patient monitoring | Solutions that allow HCW to remotely monitor users through wearable devices as well as interact with them in case of adverse events. Some solutions also support decentralized clinical trials | Doccla, AccuHealth, Everwell | TV displaying video of app instruction:  https://www.youtube.com/watch?v=dXaHrG8djV0  <https://www.youtube.com/watch?v=8DNcv3BTcuM> |
| 7 | Human resources | Online appointment scheduling | Platforms that allow users to schedule appointments with care-takers online | Doctolib, Docplanner | TV displaying video of app introduction:  <https://www.youtube.com/watch?v=rhxja4cFrhg> |
| 8 | Human resources | Medical education | Tools that facilitate knowledge about TB and increase capacity to diagnose and care for people with TB. Outreach to medical schools and District TB Units. | OppiaMobile  [Osmosis.org](http://osmosis.org/) | TV displaying founders video: https://www.youtube.com/watch?v=-G3k0jHZLWM&t=46s  TB content:<https://www.youtube.com/watch?v=6P6zBHpWiGA>  Medical edu platform:<https://www.youtube.com/watch?v=ax8EK3iTP9g> |
| 9 | Human resources | Electronic management of HR systems | Platforms that facilitate the management of health staff. They track detailed information about health workers throughout their employment, including where they are deployed, salary history, promotions and transfers, qualifications, in-service training courses, and reasons for attrition. | iHRIS | TV displaying video of app introduction:  <https://www.youtube.com/watch?v=PaHq3kJVSoU> |
| 10 | Human resources | Task shifting to the private sector | Linkage with Grab for delivery of specimens to central laboratories and pharmacies. E-voucher system for subsidized private-sector X-rays with treatment in the public sector. | Grab, Gojek | TV displaying PowerPoint presentation of PCPS |
| 11 | Financial support | Financial support programs | Existing programs that deliver cash or non-cash benefits to people living with TB or other diseases. | PASTB | TV displaying PowerPoint presentation of about PASTB |
| 12 | Financial support | Differentiating social protection | Identification of economically vulnerable individuals and linkage to financial support to cover TB treatment costs not subsidized by social health insurance | PROTECT Assessment developed into an app and linked to PASTB | TV displaying PowerPoint presentation of the development of PROTECT assessment and usage in ASSIST |
| 13 | Financial support | Digital payment platforms | Platforms that enable the digital delivery of cash and non-cash benefits as government to person transfers. Includes both frameworks / platforms to set up such transfers as well as models that already exist in other countries. | Momo, Moca, ZaloPay | TV displaying PowerPoint presentation and videos on these payment platforms |
| 14 | Financial support | Estimating TB medication stocks | Software to assist district and provincial level health authorities with creating estimations of required medications. These estimations can be used to procure TB treatment for individuals not covered by social health insurance. | OpenBoxes | TV displaying PowerPoint presentation on features of OpenBoxes  <https://openboxes.com/features/> |
| 15 | Active Case Finding | Innovative ACF efforts | Prioritization of locations and populations for cost effectiveness (ensuring high yield) | MATCH framework | TV displaying PowerPoint presentation on MATCH |
| 16 | Active Case Finding | Molecular tests | Devices and assays that are portable and may be used at the point of care (POC) to perform testing | Standard M10 MTB/NTM, specimen pooling | TV displaying PowerPoint presentation on pooling & one on Standard M10  https://www.youtube.com/watch?v=T9f7-7mJCJg&t=1s  <https://www.youtube.com/watch?v=bAFH6KmDOpo> |
| 17 | Active Case Finding | CAD tools & Digital X-Ray | Software that uses AI to analyze radiological images and identify pathologies | Vinbrain, Lunit Insight CXR | Digital X-ray machine, Lunit, and Vinbrain boxes |
| 18 | Active Case Finding | Antigen test - LAM | Urine test that tests for the antigen LAM (part of the cell wall off TB) | Biopromic third generation LAM | TV displaying video of app introduction  https://www.youtube.com/watch?v=xGPP9MiSMWA |
| 19 | Active Case Finding | AI Assisted cough analysis | Software that uses AI to analyze cough sounds to identify the probability of the user having TB and / or other conditions | Swaasa, TimBre | Phone demo using the Swaasa app  <https://healthcheck.swaasa.ai/#/> |
